# Significant Unconventional Anomalous Hall Effect in Heavy Metal/Antiferromagnetic Insulator Heterostructures

**DOI:** 10.1002/advs.202206203

**Published:** 2023-01-26

**Authors:** Yuhan Liang, Liang Wu, Minyi Dai, Yujun Zhang, Qinghua Zhang, Jie Wang, Nian Zhang, Wei Xu, Le Zhao, Hetian Chen, Ji Ma, Jialu Wu, Yanwei Cao, Di Yi, Jing Ma, Wanjun Jiang, Jia‐Mian Hu, Ce‐Wen Nan, Yuan‐Hua Lin

**Affiliations:** ^1^ School of Materials Science and Engineering Tsinghua University Beijing 100084 China; ^2^ Faculty of Materials Science and Engineering Kunming University of Science and Technology Kunming Yunnan 650093 China; ^3^ Department of Materials Science and Engineering University of Wisconsin‐Madison Madison WI 53706 USA; ^4^ Institute of High Energy Physics Chinese Academy of Sciences Beijing 100049 China; ^5^ Institute of Physics Chinese Academy of Sciences Beijing 100049 China; ^6^ State Key Laboratory of Functional Materials for Informatics Shanghai Institute of Microsystem and Information Technology Chinese Academy of Sciences Shanghai 200050 China; ^7^ CAS Center for Excellence in Superconducting Electronics (CENSE) Chinese Academy of Sciences Shanghai 200050 China; ^8^ Department of Physics Tsinghua University Beijing 10084 China; ^9^ Ningbo Institute of Materials Technology and Engineering Chinese Academy of Sciences Ningbo Zhejiang 315021 China; ^10^ Center of Materials Science and Optoelectronics Engineering University of Chinese Academy of Sciences Beijing 100049 China

**Keywords:** anomalous Hall effect, antiferromagnetic insulator, antiferromagnetic–paramagnetic phase transition, heavy metal, heterostructures

## Abstract

The anomalous Hall effect (AHE) is a quantum coherent transport phenomenon that conventionally vanishes at elevated temperatures because of thermal dephasing. Therefore, it is puzzling that the AHE can survive in heavy metal (HM)/antiferromagnetic (AFM) insulator (AFMI) heterostructures at high temperatures yet disappears at low temperatures. In this paper, an unconventional high‐temperature AHE in HM/AFMI is observed only around the Néel temperature of AFM, with large anomalous Hall resistivity up to 40 nΩ cm is reported. This mechanism is attributed to the emergence of a noncollinear AFM spin texture with a non‐zero net topological charge. Atomistic spin dynamics simulation shows that such a unique spin texture can be stabilized by the subtle interplay among the collinear AFM exchange coupling, interfacial Dyzaloshinski–Moriya interaction, thermal fluctuation, and bias magnetic field.

## Graphical abstract

An unconventional high‐temperature anomalous Hall effect was achieved in NiO‐based heavy metal/antiferromagnetic insulator heterostructures by controlling the antiferromagnetic‐paramagnetic transition. The anomalous Hall resistivity reached up to 40 nΩ cm. Atomistic spin dynamics simulations show that a noncollinear antiferromagnetic spin texture with a non‐zero net topological charge can be stabilized and cause such a significant anomalous Hall effect.

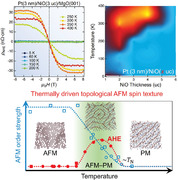

## 1 Introduction

Heavy metal (HM)/antiferromagnetic (AFM) insulator (AFMI) heterostructures are an emerging essential system for investigating the interaction between spin current and AFM order. They have the potential for applications in energy‐efficient, ultrafast, and robust spintronics devices.^[^ ^1^, ^2^, ^3^, ^4^, ^5^, ^6^, ^7^, ^8^, ^9^, ^10^ ^]^ In particular, the reflected spin current after interactions with the magnetic order at the interface can provide valuable information for determining the magnetic order, which has already been well established in the counterpart HM/ferromagnet (FM) heterostructures, such as the spin Hall magnetoresistance (SMR) and its derivative spin Hall‐anomalous Hall effect (SH–AHE).^[^ ^11^, ^12^ ^]^ Most recently, considerable attention has been paid to SMR in HM/AFMI heterostructures, which is considered a probe for current‐induced switching of the AFM order.^[^ ^2^, ^13^, ^14^, ^15^, ^16^ ^]^ Despite the extensive investigations of SMR in HM/AFMI heterostructures, the anomalous Hall effect (AHE) in such systems has not yet been subjected to the same examination, therefore, its origin, particularly whether it is caused by SMR as in HM/FM heterostructures, remains unclear.

Recently, a high‐temperature AHE was observed in HM/AFMI heterostructures, which are based on Ta, Pt, and W grown on Cr_2_O_3_ (AFMI) with an anomalous Hall resistivity (ρ_AHE_) of approximately 1 nΩ cm.^[^ ^3^, ^4^, ^5^ ^]^ Despite the controversy regarding the underlying mechanism, a ubiquitous phenomenon insensitive to the selection of the HM and crystalline orientation of AFMI was observed. That is, a superparamagnetism‐like AHE signal with a zero‐coercive field (*H*_c_) exists at temperatures significantly higher than the bulk Cr_2_O_3_ Néel temperature *T*_N_ (307 K) but disappears in the low‐temperature region (< 200 K).^[^ ^3^, ^4^, ^5^ ^]^ However, for heterostructures consisting of AFMI with significantly higher bulk *T*_N_, for example, the *α*‐Fe_2_O_3_ (*T*_N_ ≈ 950 K) and NiO (*T*_N_ ≈ 523 K), an AHE at approximately room temperature has not been reported. Thus, it is desirable to extend the AHE to other AFMI‐based heterostructures, understand its physical mechanism, and develop methods to adjust its magnitude.

In this study, we demonstrate that the AHE can be induced in HM/NiO heterostructures at elevated temperatures (up to 400 K) by controlling the AFM–paramagnetic (PM) (AFM–PM) transition of an ultrathin epitaxial NiO film. The value of ρ_AHE_ was approximately 40 nΩ cm, which is higher than the recorded ρ_AHE_ in HM/magnetic insulator heterostructures. The origin of the AHE in HM/AFMI heterostructures is attributed to the emergence of AFM spin textures with uncompensated topological charges during the AFM–PM phase transition. Moreover, the line shape of the AHE signal depends on the defect at the HM/AFMI interface, which can induce topological‐Hall‐effect‐like (THE‐like) signals based on the two‐channel AHE scenario.

## 2 Results and Discussion

**Figure 1:**
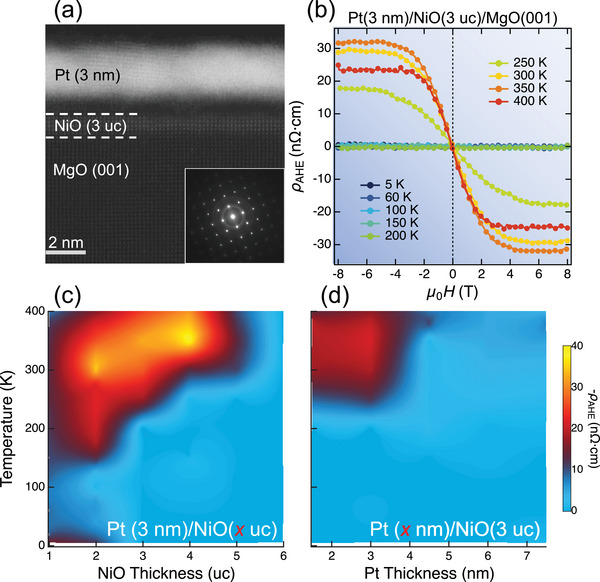
Observation of AHE in Pt/NiO/MgO heterostructures. a) The STEM of Pt(3 nm)/NiO(3 uc)/MgO(001) (the inset is the SAED pattern). b) ρ_AHE_ of the same sample at varying temperatures. c,d) Evolution of the ρ_AHE_ with temperature and thickness of NiO and Pt.

We first employed scanning transmission electron microscopy (STEM) to characterize the structural properties of the HM/NiO heterostructures with ultrathin NiO. The STEM image and selected area electron diffraction (SAED) pattern of a representative Pt(3 nm)/NiO(3 uc)/MgO(001) heterostructure are shown in **Figure** **1**a, where uc denotes unit cells (see details in Experimental Section). The NiO was fully epitaxial on the MgO(001) substrate with a rock‐salt crystalline structure, whereas the Pt overlayer was polycrystalline. The field‐dependent magnetization measurements showed no observable macroscopic ferromagnetism (see Figure S1, Supporting Information). To investigate both the Pt and NiO thickness dependence of the ρ_AHE_, we performed Hall measurements at 5–400 K, with subtraction of the linear ordinary Hall signal (Complete AHE data are presented in Figure S2, Supporting Information). A low driving current density of ≈10^2^ A cm^−2^ was used to suppress the Joule heating. The absence of an AHE in the reference sample without the NiO layer is shown in Figure S3 (Supporting Information).

A temperature‐dependent AHE was observed in the Pt(3 nm)/NiO(3 uc)/MgO(001) heterostructure (Figure 1b). The AHE was only observed at high temperatures (250–400 K). This trend is consistent with that of Cr_2_O_3_‐based HM/AFMI heterostructures.^[^ ^3^, ^4^, ^5^ ^]^ Comprehensive contour plots of ρ_AHE_ in Pt(3 nm)/NiO(*x* uc)/MgO(001) and Pt(*x* nm)/NiO(3 uc)/MgO(001) are shown in Figure 1c,d. The AHE mainly existed above 150 K, and its magnitude significantly depended on the NiO and Pt thicknesses. As the NiO thickness increased, the minimum temperature required for the appearance of ρ_AHE_ increased. For NiO thicker than 6 uc, we observed no apparent ρ_AHE_ signals within the accessible temperature (5–400 K) and field (±8 T) ranges. Figure 1d shows the decay of ρ_AHE_ as Pt becomes thicker, indicating the interfacial nature of the AHE. It is noted that a local maximum of ρ_AHE_ appeared at approximately 5 K, which could be induced by the skew scattering of electrons by localized paramagnetic centers (Giovannini–Kondo model).^[^ ^17^, ^18^ ^]^

**Figure 2:**
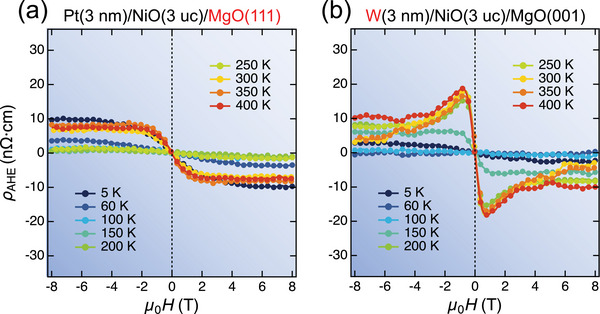
Insensitivity of AHE to AFMI orientation and HM type. a,b) ρ_AHE_ of Pt(3 nm)/NiO(3 uc)/MgO(111) and W(3 nm)/NiO(3 uc)/MgO(001) heterostructures at various temperatures.

**Figure 3:**
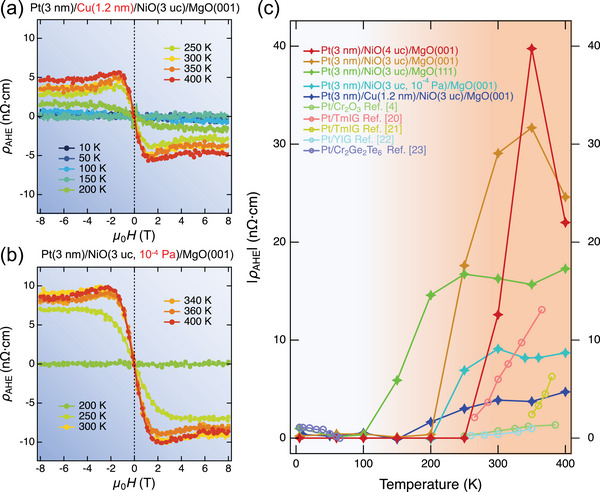
Control experimental results for AHE in HM/AFMI heterostructures. a,b) ρ_AHE_ of Pt(3 nm)/NiO(3 uc)/MgO(111) and W(3 nm)/NiO(3 uc)/MgO(001) at various temperatures. c) Temperature‐dependent saturated ρ_AHE_ from our data and reported HM/magnetic insulators.^[^ ^4^, ^20^, ^21^, ^22^, ^23^ ^]^ The four‐pointed stars represent our data, and the circles represent data from references.

As a *G*‐type collinear AFM, the superexchange interaction between Ni ions results in antiferromagnetically stacking of ferromagnetic {111} planes.^[^ ^10^ ^]^ To investigate how the crystallographic orientation of AFMI and the type of HM influence the AHE, we further measured ρ_AHE_ in the Pt(3 nm)/NiO(3 uc)/MgO(111) and W(3 nm)/NiO(3 uc)/MgO(001) heterostructures. A similar temperature‐dependent AHE behavior was observed (**Figure** **2**), indicating that the AHE was insensitive to the crystal orientation of AFMI and the type of HM. In particular, W showed the opposite sign of the spin Hall angle to Pt,^[^ ^19^ ^]^ however, the sign of AHE remained the same.^[^ ^4^ ^]^ In addition, the line shape of ρ_AHE_ for W(3 nm)/NiO(3 uc)/MgO(001) was slightly different from that of Pt(3 nm)/NiO(3 uc)/MgO(001), which will be discussed subsequently. Additional control experiments were performed to clarify the role of the HM and concomitant spin Hall effect (SHE) and the strong spin‐orbit coupling on the AHE. First, no AHE signal was observed for Ti(3 nm)/Cu(3 nm)/NiO(3 uc)/MgO(001) as expected (Figure S4, Supporting Information). Second, a weaker AHE signal was observed in Pt(3 nm)/Cu(1.2 nm)/NiO(3 uc)/MgO(001) (**Figure** **3**a), ruling out the proximity‐induced‐ferromagnetism in Pt. Finally, Pt/W/NiO/MgO(001) heterostructures were fabricated, and the competing of spin current^[^ ^19^ ^]^ initially decreased the magnitude of the AHE, and eventually reversed the polarity (sign) of the AHE with thickened Pt (Figure S5, Supporting Information). The experiments demonstrate that the SHE in HM plays a critical role, and the scattering of the spin current by AFMI (in a reflective manner) induces the AHE.The experimental results, along with the ρ_AHE_ values reported for HM/magnetic insulator heterostructures, are presented in Figure 3c. We achieved ρ_AHE_ values up to 40 nΩ cm in the Pt/NiO heterostructures, which is higher than the recorded value in HM/magnetic insulator heterostructures.^[^ ^4^, ^20^, ^21^, ^22^, ^23^ ^]^ Additionally, the scaling relation of the HM/AFMI heterostructure is shown in Figure S6 (Supporting Information), which is similar to that of the gating‐induced ferromagnetic Pt.^[^ ^24^, ^25^ ^]^

An additional THE‐like Hall effect (the bump and dip feature added to the AHE signals) was observed in W(3 nm)/NiO (3 uc)/MgO(001) and Pt/Cu(1.2 nm)/NiO (3 uc)/MgO(001) compared to Pt/NiO (3 uc)/MgO(001) (Figures 2b and 3b). From the Ellingham diagram, it can be observed that the Cu/Cu_2_O, Ni/NiO and W/WO_3_ lines are relatively close, whereas the Pt/PtO_2_ line is significantly higher than all the above lines.^[^ ^26^ ^]^ This implies that the oxygen can migrate from NiO to Cu and W to induce additional oxygen vacancies in NiO, resulting in weak ferromagnetism.^[^ ^27^, ^28^, ^29^, ^30^ ^]^ To confirm this defect‐induced THE‐like signal, we deposited a NiO layer with a significantly lower oxygen pressure to enrich the oxygen vacancies. A THE‐like signal was detected again (Figure 3b), indicating that the THE‐like signal was related to oxygen vacancy‐induced dilute ferromagnetism. Thus, the THE‐like signal can be regarded as an alternative two‐channel AHE scenario engineered by the defects.^[^ ^31^, ^32^ ^]^ One of the channels results from the AFM order, whereas the other is related to the dilute ferromagnetism, similar to the HM/FM heterostructures.^[^ ^12^, ^33^ ^]^

Next, we discuss the relationship between the *T*_N_ of the AFMI layer and the temperature‐dependent AHE behavior. The *T*_N_ of the ultrathin NiO is highly dependent on its thickness and boundary conditions. It has been reported that an ultrathin NiO film grown on MgO can have a significantly reduced *T*_N_ from its bulk value, for example, the *T*_N_ of NiO(3 uc)/MgO(001) was lower than 40 K, and its *T*_N_ could be considerably enhanced up to 390 K simply by Ag capping owing to image charge screening.^[^ ^34^ ^]^ Therefore, the *T*_N_ in the same 3 uc NiO film in our Pt/NiO/MgO(001) should also be enhanced by the Pt overlayer to induce a high‐temperature AHE. Note that the short‐range magnetic order above *T*_N_^[^ ^35^, ^36^, ^37^, ^38^ ^]^ could also support the high‐temperature AHE.

**Figure 4:**
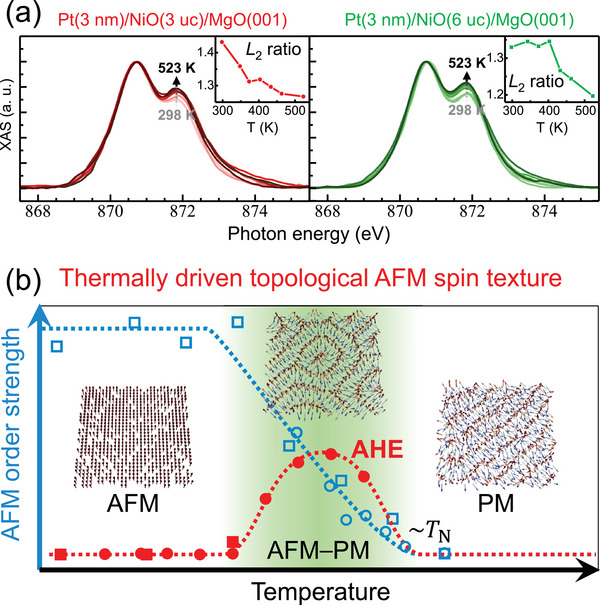
Correlation between AHE and AFM–PM transition. a) Temperature‐dependent XAS for Pt(3 nm)/NiO(3 uc)/MgO(001) and Pt(3 nm)/NiO(6 uc)/MgO(001). b) Curves are extracted from AHE (red symbols) and XAS (blue symbols) data from above two samples (circles for the sample with 3 uc NiO, and squares for that with 6 uc NiO), normalized by the putative AFM–PM transition temperature ranges. The AFM order strength is represented by the *L*_2_ ratio of XAS data. The insets show the calculated spin structures in the ultrathin NiO, represented by the distribution of Néel vector n, and the vector length is not proportional to the magnitude of the n at various temperatures.

To determine the *T*_N_ of NiO in our Pt/NiO/MgO sample, we performed temperature‐dependent X‐ray absorption spectroscopy (XAS) measurements at the Ni *L*_2_ edge. The decrease in the *L*_2_ ratio with increasing temperature indicates an AFM–PM transition.^[^ ^34^, ^39^ ^]^ The continuous decrease in the Ni *L*_2_ ratio of Pt(3 nm)/NiO(3 uc)/MgO(001) at 298–523 K indicates that this temperature range is within the continuous second‐order AFM–PM transition range of NiO (left panel of **Figure** **4**a), and the *T*_N_ should be as high as 523 K and comparable to that of the bulk NiO (Here, the *T*_N_ is defined as the temperature of the absence of the long‐range AFM order via thermal fluctuation). This indicates that Pt can impose a more pronounced effect than Ag on stabilizing the AFM order in NiO. However, for Pt(3 nm)/NiO(6 uc)/MgO(001), the Ni *L*_2_ ratio started to decline above 400 K, showing that the AFM–PM transition in this sample was initiated at 400 K. W exhibited a similar effect on 3 uc NiO, that is, the AFM–PM transition covered the temperature range of 298–523 K (Figure S7, Supporting Information). A comparison between the AHE and XAS data showed that the AHE coincided with that of the AFM–PM transition in the common temperature range of 300–400 K. Notably, the absence of AHE in Pt(3 nm)/NiO(6 uc)/MgO(001) below 400 K can be explained by the AFM–PM transition starting at temperatures higher than 400 K.

Based on the consistent temperature range between the AFM–PM transition and the AHE, the thermally softened AFM order during the AFM–PM transition plays a crucial role in the AHE of HM/AFMI heterostructures. In Cr_2_O_3_‐based HM/AFMI heterostructures, the AFM–PM transition generates the temperature‐dependent uncompensated magnetic moment on the HM/AFMI interface based on SH–AHE scenario, which is; however, questioned by X‐ray magnetic dichroism measurement.^[^ ^3^, ^4^, ^5^ ^]^ In our case, the magnetically compensated (100)‐facet and uncompensated (111)‐facet in NiO exhibited similar temperature‐dependent AHE. Thus, the conventional SH–AHE scenario based on uncompensated magnetic moments should not account for the appearance of the AHE.

Three mechanisms cause the conventional AHE: intrinsic contribution by the Berry phase and extrinsic contributions by skew scattering and side jump.^[^ ^40^, ^41^ ^]^ In this study, such an AHE in the heterostructure system occurs owing to the interaction between the spin current (SHE in HM) and the magnetic structure (magnetic insulator, in this case, AFMI). Recently, it has been reported that an AFM topological spin texture emerges around the phase‐transition temperature of Pt/*α*‐Fe_2_O_3_ heterostructures.^[^ ^42^ ^]^ A significant Fert–Levy‐type interfacial Dzyaloshinskii–Moriya interaction (DMI) exists and favors noncollinear spin textures, considering the space‐inversion symmetry breaking on the interface.^[^ ^43^ ^]^ Thus, the AFM topological spin textures could exist in HM/AFMI heterostructures at room temperature, responsible for the AHE by scattering spin‐polarized electrons with an effective field $\left\langle b_{z}\right\rangle =\sqrt{3}\phi_{0}/\left(2\lambda^{2}\right)$ for per unit topological charge, where 〈*b*_z_〉 is the out‐of‐plane effective field, *ϕ*_0_ is the unit flux and *λ* is the size of spin texture.^[^ ^44^, ^45^ ^]^

We performed atomistic spin dynamics simulations to investigate the formation of AFM topological spin textures. We found that a noncollinear AFM spin texture could emerge during the AFM–PM transition (Figure 4b) owing to the interaction among the collinear AFM exchange coupling, DMI, thermal field, and external magnetic field (see the details in Experimental Section). In the intermediate‐temperature region, the thermal energy *k*_B_ *T* is sufficiently high to destabilize the otherwise collinear AFM order (for example, the spin structure in Figure 4b with *k*_B_ *T* = 0), such that interfacial DMI can induce the noncollinear AFM spin texture. At high temperatures, the thermal perturbation dominates, yielding a PM‐like spin state. The curves in Figure 4b are guides for the eye to show the coincident temperature range between the AFM–PM transition and the AHE occurrence (the putative AFM–PM transition ranges for sample Pt(3 nm)/NiO(3 uc)/MgO(001) and Pt(3 nm)/NiO(6 uc)/MgO(001) are approximately 200–523 and 400–523 K, respectively.).

The topological charges associated with the two spin sublattices are denoted as *Q*_A_ and *Q*_B_, respectively, and can be calculated from the spatial distribution of the Néel vector (see Experimental Section and Figure S8, Supporting Information). For a canonical AFM skyrmion, the local effective fields induced by *Q*_A_ and *Q*_B_ have the same magnitude but opposite signs because *Q*_A_ + *Q*_B_ = 0 and |*Q*_A_| = |*Q*_B_| = 1 leading to the generation of a zero net effective field.^[^ ^45^ ^]^ Therefore, an AFM skyrmion cannot cause an AHE because the time‐reverse symmetry is preserved. However, applying a large out‐of‐plane magnetic field can break the time‐reverse symmetry as the two spin sublattices are canted (Figure S9, Supporting Information), yielding a non‐zero net topological charge (*Q*_A_ + *Q*_B_ ≠ 0) and hence, an AHE.

## 3 Conclusion

In this paper, we reported an unconventional high‐temperature AHE insensitive to the selection of the HM and AFMI in HM/AFMI heterostructures, which was suppressed and even eliminated in a low‐temperature region. This is explained by an extended SMR model that includes the AFM spin texture. The emergence of the AFM spin texture (e.g., skyrmion or meron) is considered to be stabilized by the DMI at the HM/AFMI interface, and can only occur at an intermediate temperature during the AFM–PM phase transition, as demonstrated via micromagnetic simulations. The THE‐like Hall effect can be triggered by controlling the fabrication conditions of the samples, which can be regarded as an additional AHE channel induced by defect‐induced weak FM. The room‐temperature AHE in AFMI heterostructures demonstrates the strong interaction between the AFM order and spin current, which aids in developing AFM‐based spintronics devices.

## 4 Experimental Section

### Sample Fabrication

The epitaxial NiO thin films were fabricated using pulsed laser deposition (PLD). The growth conditions were as follows: a growth temperature of 650 ℃ (measured using a pyrometer), oxygen background pressure of 50 mTorr, an excimer laser with a wavelength of 248 nm, a repetition rate of 3 Hz, an energy density of 1.4 J cm^−2^, and target‐substrate distance of 5.5 cm. After deposition and cooling down to room temperature, the NiO films were immediately transferred to a magnetron sputtering chamber (AJA International, Inc.) with a background pressure better than 2 × 10^−8^ torr, and heated to 150 ℃ for 15 min, to prevent possible surface gas absorption. Then, DC sputtering was then employed to deposit the Pt layer at room temperature with a power of 30 W, background Ar of 3 mTorr, resulting in a deposition rate of 2.6 nm min^‐1^. The W and Cu were deposited under the same conditions at deposition rates of 1.3 and 3.6 nm min^‐1^, respectively.

### Structural, Electrical, and Magnetic Properties Characterization

The structural properties were characterized using a high‐resolution X‐ray diffractometer (XRD, Malvern Panalytical). The magnetic hysteresis loop was measured using a superconducting quantum interference device (MPMS, Quantum Design). The transport properties were measured using the van der Pauw method in a Physical Property Measurement System (PPMS, Quantum Design DynaCool system). The measured Hall signals were first treated using an asymmetry procedure, and the ordinary Hall signals were then subtracted. Cross‐sectional high‐resolution transmission electron microscopy samples were prepared using a focused ion beam (FIB, Zeiss Auriga) with Ga^+^ ions.

### X‐Ray Absorption Spectroscopy

Element‐specific X‐ray absorption spectroscopy (XAS) was measured using total electron yield (TEY) at Beamline BL02B02 of the Shanghai Synchrotron Radiation Facility (SSRF) and Beamline 4B9B of the Beijing Synchrotron Radiation Facility (BSRF). To evaluate the magnetic properties of these NiO thin films, we utilized the magnetic linear dichroism (MLD) effect in the Ni *L*_2_ XAS. The normal incidence of X‐rays resulted in the electric field component of X‐rays being parallel to the sample plane. The sample temperature was controlled by laser heating the sample holder. A linear background connecting raw data points at 870.8 and 871.8 eV was first subtracted from the raw data, and the spectra were normalized from 0 to 1. The *L*_2_ ratio was defined as the normalized peak intensity at 871.8 eV.

### Atomistic Spin Dynamics Simulations

Micromagnetic simulations were performed to simulate the spin structure in the AFM NiO with interfacial DMI. The total Hamiltonian of the AFM thin‐film H included the Hamiltonians of the exchange interaction $H_{\mathrm{exch}}$, uniaxial anisotropy $H_{\mathrm{anis}}$, external fields $H_{\mathrm{ext}}$, and the Hamiltonian of the interfacial Dyzaloshinskii–Moriya interaction (DMI) $H_{\mathrm{DMI}}$, expressed as follows:

(1) $$\begin{array}{ccc} H & = & H_{\mathrm{exch}}+H_{\mathrm{anis}}+H_{\mathrm{ext}}+H_{\mathrm{DMI}}=-\sum_{<k,l>}JS_{k}\cdot S_{l}-\sum_{k}K_{a}{\left(S_{k}\cdot n_{e}\right)}^{2}\\ & & +\;\sum_{<k,l>}D_{kl}\cdot \left(S_{k}\times S_{l}\right) \end{array}$$

where $S_{k}$ and $S_{l}$ are the orientation vectors of the local spin and neighboring spins, respectively; *J* is the exchange constant, *K*_*a*_ is the uniaxial anisotropy constant, $n_{e}$ is the uniaxial easy axis, and $D_{kl}$ is the DMI constant.

In the simulation, a 2D system of 64 × 64 grids was considered in which each grid with a size of 1 nm × 1 nm, contained two neighboring spins. By linearly combining $S_{m}$ and $S_{n}$ inside the same gird, the net magnetization vector $m=(S_{m}+S_{n})/2$ and the Néel vector $n=(S_{m}-S_{n})/2$ were defined. The expression of the free energy was derived by substituting $S_{m}$ and $S_{n}$ with m and n, respectively, in the total Hamiltonian. Under the continuum approximation^[^ ^46^ ^]^, the total free energy is expressed as follows:

(2) $$\begin{array}{ccc} E_{\mathrm{tot}} & = & \int dV\left\{A^{*}\left[{\left(\partial_{x}n\right)}^{2}+{\left(\partial_{y}n\right)}^{2}\right]-K{\left(n\cdot n_{e}\right)}^{2}\right.\\ & & \left.+\;D\left[n_{z}\left(\nabla \cdot n\right)-\left(n\cdot \nabla \right)n_{z}\right]-H_{\mathrm{ext}}\cdot n\right\} \end{array}$$

where A* = −2.42 pJ m^−1^, is the continuum‐scale exchange constant, which was converted from the AFM exchange interaction *J* = −19.01 meV.^[^ ^47^ ^]^ We assumed the existence of a perpendicular magnetic anisotropy (i.e., $n_{e}//z$) in the ultrathin (3 uc) NiO owing to the interfacial symmetry breaking, with a continuum anisotropy constant *K* = 1.5 × 10^5^ J m^−3^(which is a typical number utilized in existing micromagnetic simulation investigations of AFM skyrmions)^[^ ^48^ ^]^, D* = −3.5 × 10^−3^ J m^−2^ is the continuum DMI constant, which is a value calculated from the first‐principles in a similar NiO/Au system.^[^ ^49^ ^]^

The free energy expression of the Néel vector n in Equation 2 is the same as that of the magnetization in the ferromagnetic materials. Therefore, we performed micromagnetic simulations using the open‐source software MuMax3^[^ ^50^ ^]^ to calculate the equilibrium configuration of the Néel vector. The evolution of n was determined by solving the Landau–Lifshitz–Gilbert (LLG) equation,

(3) $$\frac{\partial n}{\partial t}=-\frac{\gamma_{0}}{1+\alpha^{2}}\left(n\times H_{\mathrm{eff}}+\alpha n\times n\times H_{\mathrm{eff}}\right)$$

where *α* = 0.01 is the Gilbert damping coefficient^[^ ^49^ ^]^ and γ_0_ is the gyromagnetic ratio; $H_{\mathrm{eff}}=-\frac{1}{\mu_{0}M_{s}}\frac{\delta E_{\mathrm{tot}}}{\delta n}$ is the effective field, where *μ*_0_ is the vacuum permeability, amd *M*_*s*_ = 6.2 × 10^5^ A m^−1^ is the saturation magnetization of NiO.^[^ ^49^ ^]^

The influence of thermal fluctuations was modeled by adding a thermal fluctuation field $H_{\mathrm{therm}}$ to $H_{\mathrm{eff}}$, expressed by $H_{\text{therm}\;}=\eta \sqrt{\frac{2\alpha k_{B}T}{\mu_{0}M_{s}\gamma_{0}\Delta V\Delta t}}$, where *k*_*B*_ *T* is the Boltzmann constant, *T* is the temperature, Δ*V* is the volume of each simulation cell, and Δ*t* is the time interval in a real unit. $\eta =\eta \left(r,t\right)=\left(\eta_{x},\eta_{y},\eta_{z}\right)$ is a white‐noise distributed stochastic vector uncorrelated in space and time. The temporal mean value of *η*_*i*_(*i* = *x*, *y*, *z*) was zero. The LLG equation was solved using fourth‐order Runge–Kutta methods, with a discretized time interval of 20 fs. A uniform distribution of n in the *z*‐direction was used as the initial state for the low‐temperature region (< 8 K), and an artificially generated single AFM skyrmion was used for the higher‐temperature region (> 8 K). The equilibrium distribution of n was assumed to be reached when the total free energy density *E*_tot_ no longer changes significantly with time.

The topological charge of the two sublattices of n in the equilibrium state, *Q*_A_ and *Q*_B_ were calculated as follows to quantify the topological state of the AFM thin film^[^ ^51^ ^]^:

(4) $$\begin{array}{cc} & Q_{A}=\frac{1}{8\pi }\int_{\text{site A}}q_{ijk};\\ & Q_{B}=\frac{1}{8\pi }\int_{\text{site B}}q_{ijk} \end{array}$$

where *q*_*ijk*_ is calculated for each unique triangle with grids *i*, *j*, and *k* as vertices using Equation 5

(5) $$\tan\left(\frac{q_{ijk}}{2}\right)=\frac{n_{i}\cdot \left(n_{j}\times n_{k}\right)}{1+n_{i}\cdot n_{j}+n_{i}\cdot n_{k}+n_{j}\cdot n_{j}}$$

The equilibrium state of n was determined based on the values of *Q*_A_ and *Q*_B_. If *Q*_A_ + *Q*_B_ = 0, the distribution of n is identified as a collinear distribution, otherwise, it is a topological AFM spin texture. If *Q*_A_ ≠ *Q*_B_, there is no evident ordered spin texture.^[^ ^46^ ^]^

## Conflict of Interest

The authors declare no conflict of interest.

## Supporting information

Supporting Information

## Data Availability

The data supporting the findings of this study are available within the paper and its Supporting Information, and also from the corresponding authors upon reasonable request.
