# Supplementary material for: Significant Unconventional Anomalous Hall Effect in Heavy Metal/Antiferromagnetic Insulator Heterostructures
Source: Adv Sci (Weinh). 2023 Jan 26;10(8):2206203. doi: 10.1002/advs.202206203 (PMC10015866; doi:10.1002/advs.202206203)
Supplement: Supplementary file 1 — Supporting Information [file ADVS-10-2206203-s001.pdf]

ADVANCED SCIENCE
Open Access

Supporting Information

for *Adv. Sci.*, DOI 10.1002/advs.202206203

Significant Unconventional Anomalous Hall Effect in Heavy Metal/Antiferromagnetic Insulator Heterostructures

Yuhan Liang, Liang Wu, Minyi Dai, Yujun Zhang, Qinghua Zhang, Jie Wang, Nian Zhang, Wei Xu, Le Zhao, Hetian Chen, Ji Ma, Jialu Wu, Yanwei Cao, Di Yi, Jing Ma, Wanjun Jiang, Jia-Mian Hu, Ce-Wen Nan* and Yuan-Hua Lin**

Supporting Information

Significant Unconventional Anomalous Hall Effect in Heavy Metal/Antiferromagnetic Insulator Heterostructures

Yuhan Liang,[1,*] Liang Wu,[2,†] Minyi Dai,[3] Yujun Zhang,[4] Qinghua Zhang,[5] Jie Wang,[2] Nian Zhang,[6,7] Wei Xu,[4] Le Zhao,[8] Hetian Chen,[1] Ji Ma,[2] Jialu Wu,[1] Yanwei Cao,[9,10] Di Yi,[1] Jing Ma,[1] Wanjun Jiang,[8] Jia-Mian Hu,[3] Ce-Wen Nan,[1,‡] and Yuan-Hua Lin[1,§]

[1]*School of Materials Science and Engineering, Tsinghua University, Beijing, 100084, China*
[2]*Faculty of Materials Science and Engineering, Kunming University of Science and Technology, Kunming, 650093, Yunnan, China*
[3]*Department of Materials Science and Engineering, University of Wisconsin-Madison, Madison, WI, USA*
[4]*Institute of High Energy Physics, Chinese Academy of Sciences, Beijing 100049, China*
[5]*Institute of Physics, Chinese Academy of Sciences, Beijing 100049, China*
[6]*State Key Laboratory of Functional Materials for Informatics, Shanghai Institute of Microsystem and Information Technology, Chinese Academy of Sciences, Shanghai 200050, China*
[7]*CAS Center for Excellence in Superconducting Electronics (CENSE), Chinese Academy of Sciences, Shanghai 200050, China*
[8]*Department of Physics, Tsinghua University, Beijing 100084, China*
[9]*Ningbo Institute of Materials Technology and Engineering, Chinese Academy of Sciences, Ningbo 315201, China*
[10]*Center of Materials Science and Optoelectronics Engineering, University of Chinese Academy of Sciences, Beijing 100049, China*

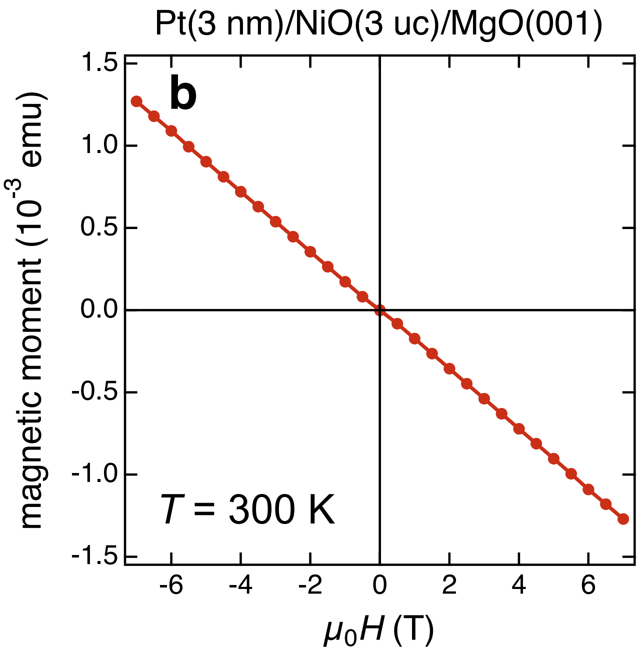

Figure S1. M–H of Pt(3 nm)/NiO(3 uc)/MgO(001) at 300 K.

* These authors contributed equally
† These authors contributed equally; liangwu@kust.edu.cn
‡ cwnan@tsinghua.edu.cn
§ linyh@tsinghua.edu.cn

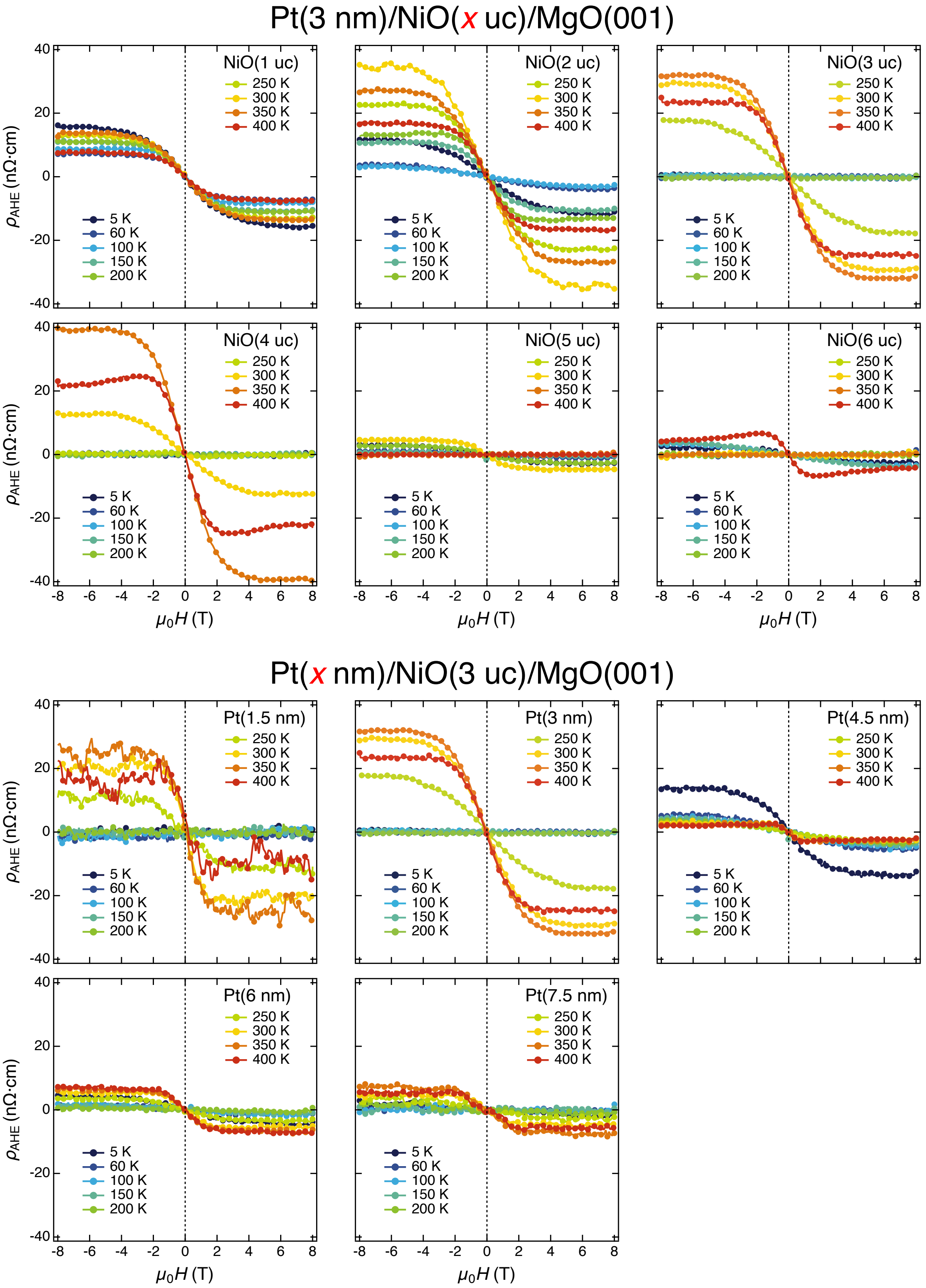

Figure S2. Detailed AHE data with varying the thickness of NiO (lower panel) and Pt (upper panel), from which the Fig 1(c) and (d) were obtained. The AHE of Pt(3 nm)/NiO(3 uc)/MgO(001) was repeated to make each panel more self-contained.

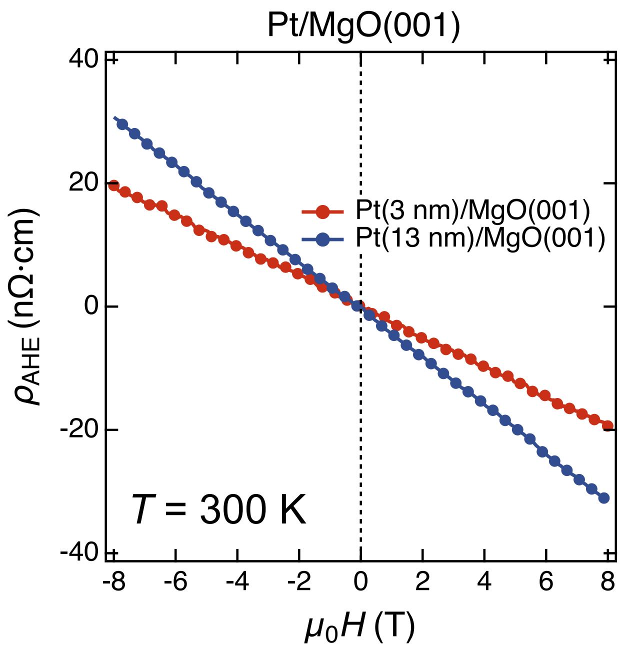

Figure S3. Hall effect of Pt/MgO(001) at 300 K.

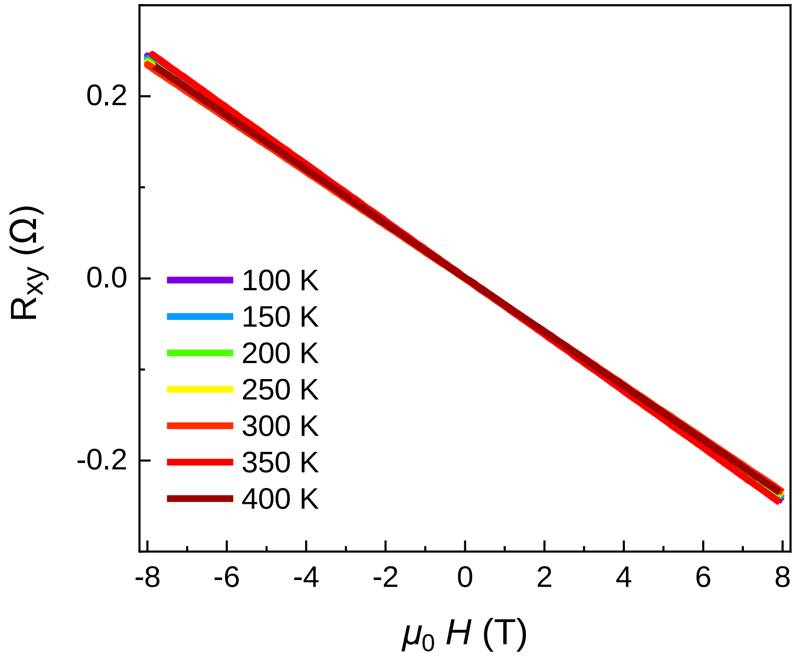

Figure S4. The temperature dependent AHE results of Ti/Cu/NiO/MgO(001) heterostructure.

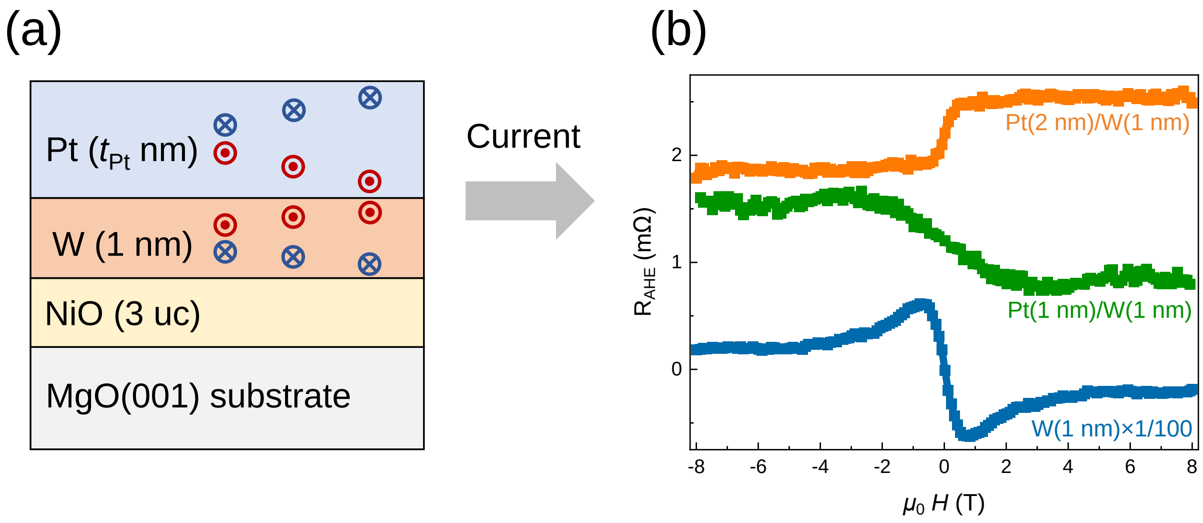

Figure S5. The reverse of unconventional AHE by competition of spin current. (a) The schematic diagram of Pt/W/NiO/MgO(001) heterostructures. (b) The reverse of AHE with the increasing of Pt layer thickness.

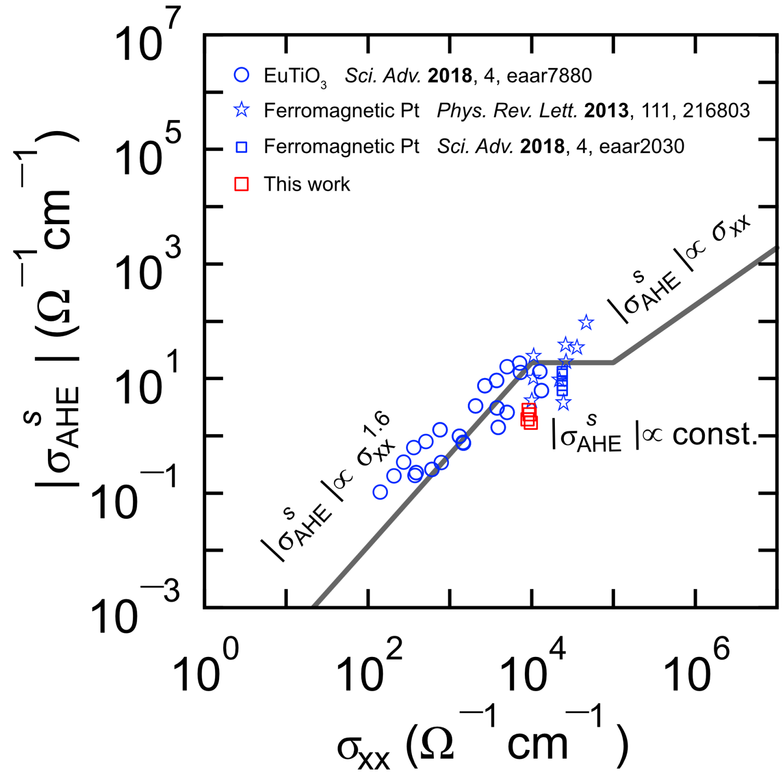

Figure S6. The scaling law of AHE in Pt/NiO/MgO(001) heterostructure compared to related works.

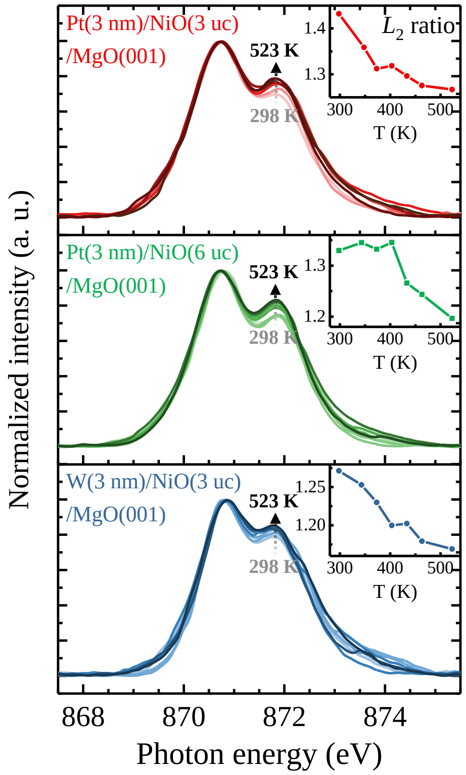

Figure S7. XAS of Ni L_2 edge for Pt(3 nm)/NiO(3 uc)/MgO(001), Pt(3 nm)/NiO(6 uc)/MgO(001) and W(3 nm)/NiO(3 uc)/MgO(001) at varying temperatures. Insets: temperature-dependent Ni L_2 ratio.

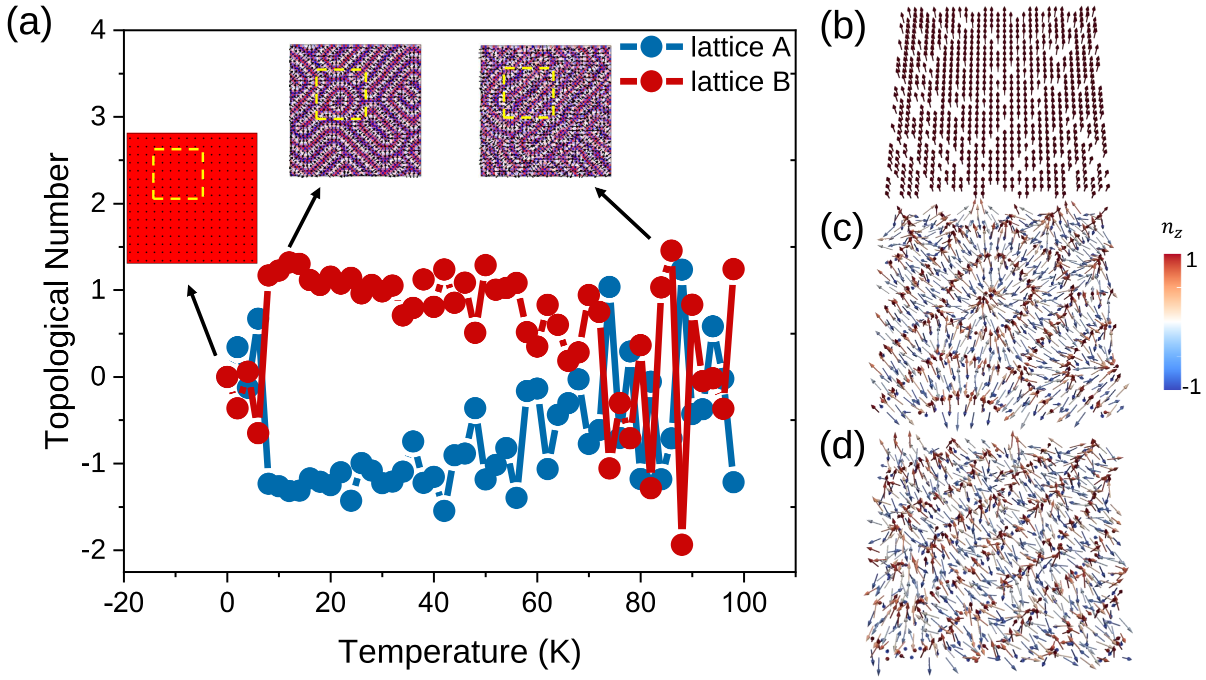

Figure S8. The calculated topological number in the absence of external field. (a) The temperature-dependent topological number of sublattice A and sublattice B. The enlarged area donoted by yellow dash box in (a) is shown in 0 K (b), 10 K (c) and 90 K (d), respectively.

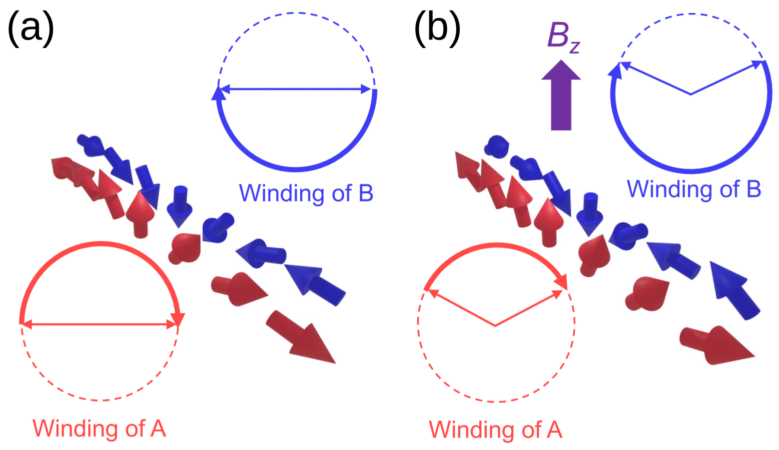

Figure S9. (a) The compensated topological charge (b) The uncompensated topological charge induced by external field.
